# Assessing the Skeletal Muscle Pump During Lower Limb Counterpressure: Lags and Causality in Cardiovascular Regulation

**DOI:** 10.1002/jcsm.70019

**Published:** 2025-07-31

**Authors:** Silvin P. Knight, Eoin Duggan, Feng Xue, Roman Romero‐Ortuno

**Affiliations:** ^1^ FRAILMatics Research Group, Discipline of Medical Gerontology, School of Medicine Trinity College Dublin Dublin Ireland; ^2^ Falls and Syncope Unit (FASU), Mercer’s Institute for Successful Ageing (MISA), St James’s Hospital Dublin Ireland

**Keywords:** cardiovascular dynamics, electromyography, near‐infrared spectroscopy, orthostatic haemodynamics, physical counterpressure manoeuvres, skeletal muscle pump

## Abstract

**Background:**

Orthostatic hypotension (OH) is a prevalent condition among older adults, characterised by a sudden drop in blood pressure upon standing, often leading to dizziness and increased risk of falls, morbidity and mortality. The skeletal muscle pump is thought to be important in maintaining venous return and stabilising blood pressure during postural changes, especially during the performance of physical counterpressure manoeuvres (PCMs). This study investigated the temporal relationships between thigh muscle activation, thigh haemoglobin concentration and cardiovascular parameters (heart rate [HR], stroke volume [SV] and total peripheral resistance [TPR]) to understand the muscle pump's role in haemodynamic regulation while performing supine and standing PCMs.

**Methods:**

Twenty‐two participants (mean age 70.4 ± 5.2 years) were assessed at the Falls and Syncope Unit at St. James's Hospital, Dublin, Ireland. Each underwent an active stand test, around which participants were asked to squeeze their thigh muscles as hard as possible for 10 s, firstly while in the supine position, and secondly after standing. Surface electromyography (EMG), near‐infrared spectroscopy (NIRS) and non‐invasive digital artery photoplethysmography were used to continuously monitor thigh muscle activation, muscle haemoglobin concentration and cardiovascular function, respectively. Cross‐correlation and Granger causality analyses were conducted to determine the temporal and causal relationships between the signals.

**Results:**

Cross‐correlation analysis revealed significant temporal relationships between muscle activation and cardiovascular parameters during PCMs. Specifically, HR, TPR and SV lagged EMG by approximately 1–3, 6–9 and 8–10 s, respectively. Mean peak cross‐correlation coefficients during the standing PCM were 0.609 for EMG to HR, 0.516 for EMG to TPR and 0.564 for EMG to SV. Granger causality tests indicated that muscle activation significantly predicted changes in SV, HR and TPR, with causality proportions increasing during PCMs (e.g., SV to EMG: 9.1% during supine rest; EMG to SV: 63.6% during standing PCM). Notably, inter‐individual variability was observed, with peak CCs for EMG to SV ranging from 0.251 to 0.849, and lag times from −21.9 to 13.4 s during standing PCM.

**Conclusions:**

These findings underscore the role of the skeletal muscle pump in modulating venous return and cardiac output during PCMs. The study provides a novel methodological framework for assessing skeletal muscle pump function and its impact on cardiovascular dynamics. By understanding the temporal interplay between muscle activation and cardiovascular responses, we can develop effective strategies to improve cardiovascular stability and potentially prevent OH. Future research should validate these findings in larger, more diverse cohorts and explore long‐term adaptations to targeted interventions.

## 1 Introduction

Orthostatic hypotension (OH) is a common and clinically significant condition, particularly among older adults, characterised by a sudden drop in blood pressure upon standing. This condition often leads to dizziness, light‐headedness, and increased risk of falls, contributing to morbidity and mortality in the older population [1]. The skeletal muscle pump is thought to play an important role in maintaining venous return and stabilising blood pressure during postural changes, especially during the performance of physical counterpressure manoeuvres (PCMs), which are deliberate and controlled ‘muscle squeezes’ to improve orthostatic tolerance [2].

Historical insights into the effects of gravity on circulation were reported as far back as 1897 by Hill and Bernard [3]. Later developments by Hellebrandt et al. in the 1930s–1940s first highlighted the significance of the skeletal muscle pump in the prevention of OH, or ‘gravity shock’ as it was termed at the time [4]. Transitioning to and maintaining stable upright posture involves complex physiological interactions that manage blood redistribution due to gravity [5]. When standing, blood pools in the veins of the lower body, reducing central venous pressure. The carotid and cardiopulmonary baroreceptors compensate for this drop in pressure reducing cardiac vagal activity and increasing sympathetic activity leading to an increase in heart rate (HR) and total peripheral resistance (TPR) [6]. However, this effect is short‐lived, as the baroreflex alone cannot sustain blood pressure regulation under prolonged orthostatic stress [7]. It is thought that during muscle contraction, particularly in the thighs, increased intramuscular pressure facilitates the expulsion of blood from the muscle's veins, aiding venous return [8]. This contraction‐induced pressure gradient assists in pushing blood towards the heart, increasing preload, and subsequently stroke volume (SV). However, the temporal dynamics of these interactions, particularly the lag between muscle activation and resultant cardiovascular responses, remain poorly understood. Identifying these temporal relationships can provide deeper insights into the muscle pump's role in haemodynamic regulation and inform the design of targeted therapeutic strategies.

Technological advancements in continuous non‐invasive physiological monitoring have enabled more precise measurement of muscle and cardiovascular function. Surface electromyography (EMG) allows for the continuous and highly detailed assessment of muscle activation, while near‐infrared spectroscopy (NIRS) provides real‐time data on haemoglobin concentration in the muscle tissue. Coupling and synchronising these modalities with non‐invasive beat‐to‐beat cardiovascular measurements using digital artery photoplethysmography offers a comprehensive way to investigate the interactions between muscle activity and cardiovascular responses. Integrating these technologies offers an opportunity to identify precise mechanisms by which the muscle pump influences cardiovascular dynamics during PCMs, which is still poorly documented in the literature [9, 10].

By examining the time delay, or lag, between different continuous physiological signals, it is possible to infer the sequential order of events and the strength of their relationships. Cross‐correlation, a specific method within lag analysis, measures the degree to which two signals are correlated as a function of the time lag between them [11]. This technique is particularly useful in physiological studies, where the synchronisation and timing between different body systems is of interest, as is the case for the present study. For instance, the time lag between the onset of muscle contractions and changes in SV could provide a metric for the efficiency of an individual's muscle pump function.

Understanding causality in physiological systems goes beyond mere correlation; it involves determining whether one signal can predict another. Granger causality is a statistical method used to assess whether one time series can significantly forecast another, thereby suggesting a directional influence. This technique is based on the premise that if a signal *X* Granger‐causes a signal *Y*, then past values of *X* contain information that helps predict *Y* beyond the information contained in past values of *Y* alone [12, 13]. Granger causality has been widely applied in various fields, including neuroscience and cardiovascular research, to decipher complex interactions and directional influences among multiple physiological signals [14, 15, 16, 17, 18].

Several small‐scale studies have applied causality methods to cardio‐postural interactions during standing. For example, Verma et al. used convergent cross mapping to examine the causal influence between calf EMG activity and systolic blood pressure in older stroke survivors versus healthy controls during quiet standing, finding that blood pressure exerted greater influence on muscle activation after stroke despite similar baroreflex sensitivity [19]. In a companion analysis, the same group applied transfer entropy to blood pressure waveforms, centre‐of‐pressure sway, and EMG in young adults, showing that blood pressure primarily drove postural sway metrics while muscle activity also influenced sway [20]. A subsequent convergent cross mapping study extended this to examine the bidirectional coupling among systolic pressure, EMG, and resultant centre‐of‐pressure, revealing stronger causal effects from muscle activity to both blood pressure and postural sway than in the reverse direction [21]. In 2019, the same group quantified age‐related changes, demonstrating that both the cardiac baroreflex (systolic pressure's effect on heart period) and the muscle‐pump baroreflex (systolic pressure's effect on EMG impulse) are significantly reduced in older compared to younger adults [22]. While these investigations clarify causal loops under passive or postural stress, none have synchronised surface EMG, NIRS‐derived haemoglobin concentration, and beat‐to‐beat HR, SV and TPR during deliberate PCMs in older adults.

The present study aimed to employ a multimodal approach to investigate the temporal relationships between thigh muscle activation, thigh haemoglobin concentration and cardiovascular parameters during orthostatic PCMs in a pilot sample of older adults undergoing an AS test. By analysing data from various physiological signals during supine rest and two distinct PCMs, we sought to elucidate the dynamic interplay between the skeletal muscle pump activation and cardiovascular responses. We hypothesised that muscle activation during PCMs, during both lying and standing, would show significant predictive relationships with changes in SV, HR and TPR, with distinct temporal lags indicating the muscle pump's role in cardiovascular regulation.

## 2 Methods

### 2.1 Participants

Twenty‐two volunteer participants were recruited from a local healthy ageing group and assessed at the Falls and Syncope Unit (FASU) at Mercer's Institute for Successful Ageing (MISA) in St James's Hospital, Dublin, Ireland. Criteria for inclusion in the study were being aged 50 years or older, capable of providing written informed consent, able to mobilise independently (with or without aid), and able to transfer independently (or with minimal assistance of one person) from lying to standing. Participants with a history of neurogenic OH or a cardiac pacemaker were not included.

### 2.2 Experimental Protocol

The AS test consisted of a supine resting phase of ~10 min, followed by the participant standing as quickly as possible and remaining standing quietly for ~4 min. The protocol incorporated two PCMs, where participants were asked to squeeze their thigh muscles as hard as possible and to keep the muscles squeezed for 10 s. The first PCM was conducted ~5 min into the supine rest and the second 3 min after standing. Prior to the first squeeze, the researchers demonstrated the correct procedure for performing the thigh squeeze, and each participant was allowed at least one practice squeeze to ensure proper technique (these practice runs were not recorded). All measurements were carried out in a comfortably lit room at an ambient temperature between 21 and 23 °C.

### 2.3 Cardiovascular Measurements

**FIGURE 1:**
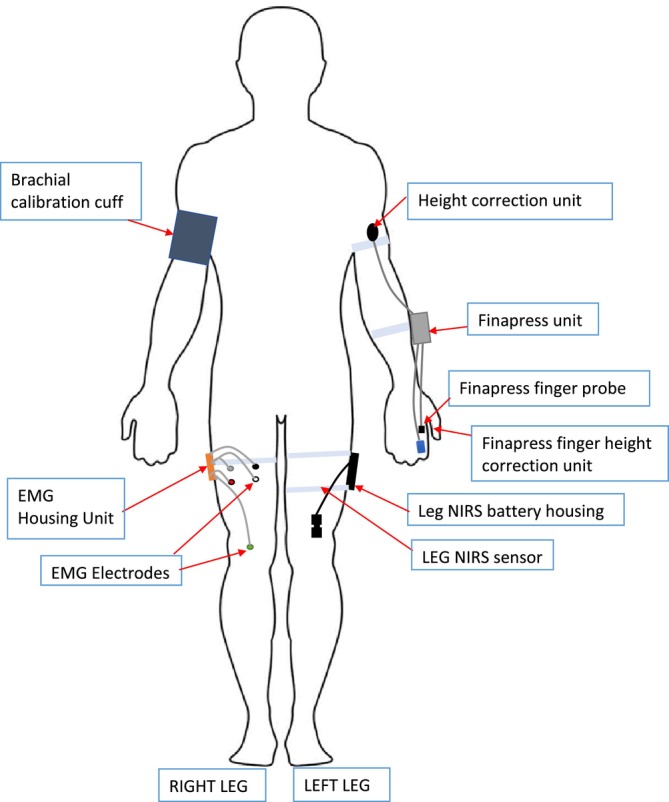
Schematic of the experimental device setup. Abbreviations: electromyography (EMG); near‐infrared spectroscopy (NIRS).

Participants began the assessment with the affixing of a digital artery photoplethysmograph to the index finger of the left hand (Finapres Nova device, Finapres Medical Systems BV, Enschede, the Netherlands), as shown in Figure 1. Height changes were adjusted for using the built‐in height sensor on the Finapres device. Device‐derived beat‐to‐beat signals for the haemodynamic parameters were collected, including HR, SV and TPR. For descriptive purposes, mean arterial pressure (MAP) and cardiac output (CO) data were also collected.

### 2.4 Thigh Muscle Measurements

Muscle activation measurements were made using a surface EMG device (Shimmer 3; Shimmer Sensing, Dublin, Ireland), with a sampling rate of 1024 Hz. A pair of self‐adhesive Ag/AgCl electrodes of 24 mm diameter (Kendall H124SG, Cardinal Health, Waukegan, Illinois, United States) were positioned over the midline of the rectus femoris muscle of the right thigh as per the Surface EMG for a Non‐Invasive Assessment of Muscles (SENIAM) guidelines [23], at a point halfway between the anterior superior iliac spine and the superior patella. A near‐infrared spectroscopy (NIRS) device (Portalite; Artinis Medical Systems, Elst, The Netherlands) was employed to measure haemoglobin concentration changes in the left thigh, as also shown in Figure 1, at a sampling rate of 50 Hz. This NIRS device uses three transmitters and one receiver, with each transmitter emitting two different wavelengths of light (760 nm and 850 nm) that propagate through the tissue to a depth of approximately 2–3 cm and are absorbed at different rates by oxygenated haemoglobin (O2Hb) and deoxygenated haemoglobin (HHb). O2Hb and HHb concentrations were recorded, and total haemoglobin concentration (Hb) was calculated as the sum of O2Hb and HHb.

### 2.5 Other Measures

Participants' medical history and medication use were obtained from self‐report, along with information about smoking and alcohol use. Number of cardiovascular conditions counted hypertension, hypercholestrolaemia, ischaemic heart disease, valvular heart disease and/or arrhythmia. Cardiovascular medication use was coded as ‘Yes’ if the participant was taking one or more of the following medications (coded using the Anatomical Therapeutic Chemical Classification (ATC)): anti‐arrhythmics (ATC C01), anti‐hypertensives (ATC C02), diuretics (ATC C03), vasodilators (ATC C04), beta‐blocking agents (ATC C07), calcium channel blockers (ATC C08) or agents acting on the renin‐angiotensin system (ATC C09). Psychotropic medication use was coded as ‘Yes’ if the participant was taking one or more of the following: anti‐epileptics (ATC N03A), anti‐psychotics, anxiolytics, hypnotics or sedatives (ATC N05) or anti‐depressants (ATC N06A). Height was measured with the Seca 213 stadiometer (Seca, Hamburg, Germany) to the nearest 0.001 m with the participant barefoot. Weight was measured using the TANITA DC‐430 MAP device. BMI was calculated from the formula weight [kg]/(height [m]^2^.

### 2.6 Data Processing

**FIGURE 2:**
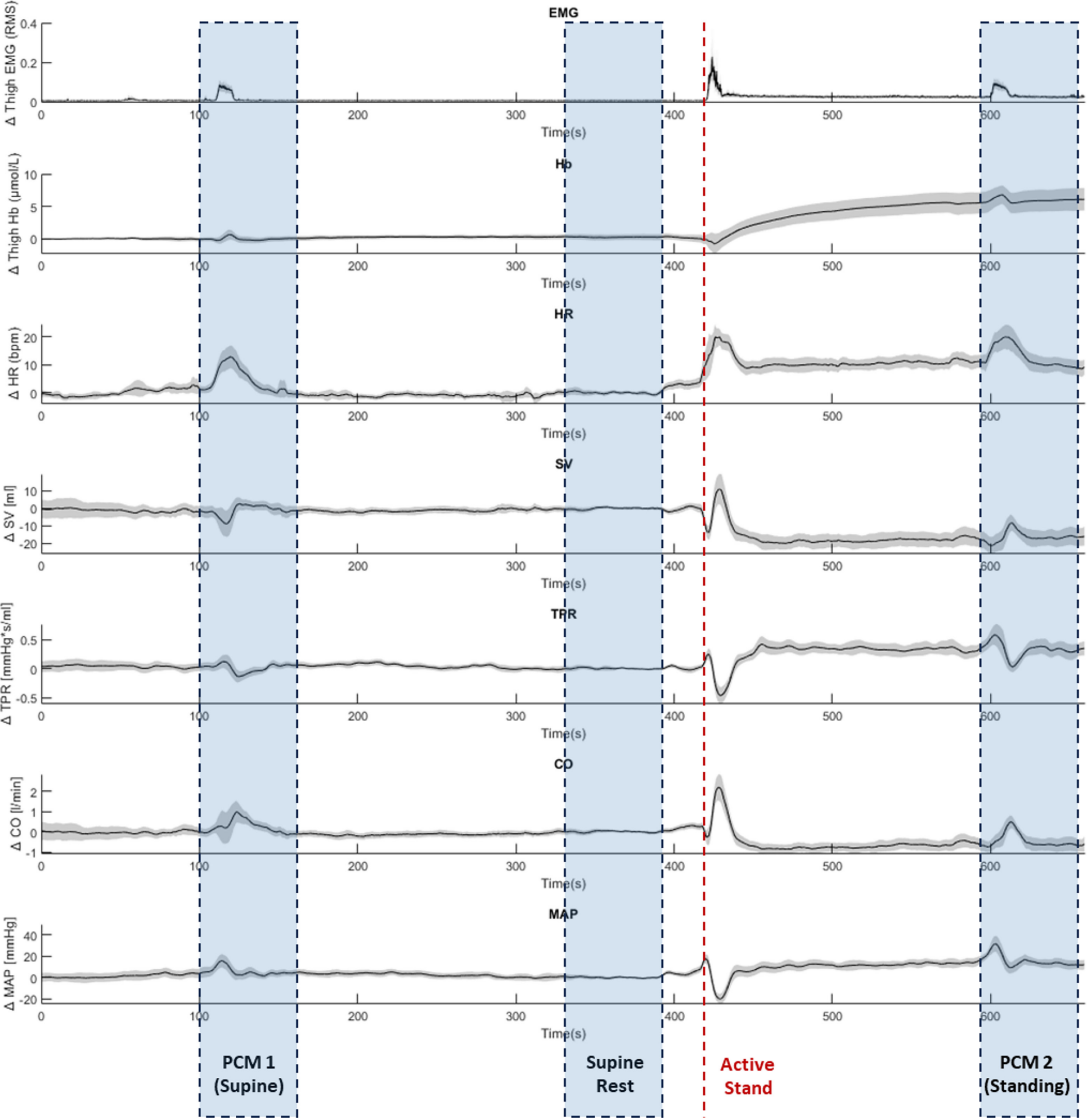
Mean signals in the 22 participants showing change from baseline. Ninety‐five percent confidence intervals are also shown. Abbreviations: electromyography (EMG); total haemoglobin concentration (Hb); heart rate (HR); stroke volume (SV); total peripheral resistance (TPR); physical counterpressure manoeuvre (PCM); cardiac output (CO); mean arterial pressure (MAP).

All signal processing was performed using MATLAB 2023a (The MathWorks Inc., Natick, Massachusetts, United States). Signals were precisely aligned using synchronised markers, set on each device during data acquisition. Three regions of interest (ROIs) were selected from the data: ‘supine rest’, taken as the one‐minute section of data 30 s before the stand; ‘PCM 1’ and ‘PCM 2’, both 1‐min sections of data starting 10 s before each respective squeeze. ROI selection is illustrated in Figure 2.

All signals were resampled to a uniform rate of 10 Hz prior to analysis [8]. NIRS data were downsampled from 50 to 10 Hz by binning each consecutive sets of 5 data points together and taking the mean. Linear interpolation was used to resample the beat‐to‐beat data (HR, SV and TPR) to 10 Hz. For the EMG data, the downsampling from 1024 to 10 Hz was achieved by computing the root mean square (RMS) of 102‐sample windows, effectively reducing the data's temporal resolution while preserving its essential features. We took the RMS to make the EMG signal more comparable to the other signals, as the RMS value provides a measure of signal amplitude that is less affected by high‐frequency noise and is more representative of the overall signal power. Moreover, previous studies have shown that the cardio‐postural control loop interaction exists in the lower frequency range (< 1 Hz) [8, 24]. This was particularly important since the experimental paradigm under investigation involved inducing a 10‐s‐long change in signal and observing its propagation across signals measured in other systems, and as such high frequency data was not of interest. To ensure comparability between signals, all signals were also normalised using the ‘zscore’ method, which centres the data to have mean = 0 and scales it to have standard deviation = 1.

### 2.7 Stationarity Testing and Granger Causality

To ensure the validity of Granger causality, all ROI timeseries were first assessed for stationarity using two complementary unit‐root tests. The augmented Dickey–Fuller (ADF) test was applied to each ROI and variable, with the null hypothesis that a series is non‐stationary (i.e., contains a unit root) and the alternative of stationarity [25]. In parallel, the Kwiatkowski–Phillips–Schmidt–Shin (KPSS) test was used with the null of stationarity and the alternative of a unit root to guard against type I errors inherent to a single test [26]. If *both* ADF and KPSS indicated non‐stationarity for a given series (*p* > 0.05 for ADF; *p* ≤ 0.05 for KPSS), that series was first‐differenced and retested to confirm stationarity. Differencing was only applied to non‐stationary series; stationary series were left unchanged, preserving their original frequency content for causality testing.

For each participant and ROI, pairwise Granger causality was then estimated on the stationary data using a vector autoregressive (VAR) model of order p chosen by the Akaike information criterion (AIC) [27]. We tested the hypothesis that past values of series *X* improve the prediction of series *Y* over and above past values of *Y* alone (and vice versa), yielding F‐statistics and associated p‐values. A result was deemed significant if *p* ≤ 0.05. To address potential biases from pre‐differencing, we conducted a secondary analysis using the Toda–Yamamoto procedure, which augments the VAR lag length by the maximum integration order (d_max_) of the two series—thus providing valid causality tests even in the presence of non‐stationary but not cointegrated data [28]. Finally, the proportion of participants exhibiting significant causality in each ROI was reported, providing a robust measure of directional coupling that accounts for stationarity requirements.

### 2.8 Cross‐Correlation Analysis

Cross‐correlation analysis was conducted to identify the temporal lags between EMG, NIRS and cardiovascular parameters (SV, HR, TPR). This method involved computing the cross‐correlation function (CCF) between EMG and NIRS signals and the three cardiovascular signals (SV, HR, TPR) at varying the time shift between the sets of two variables. Plots of the CCFs versus all possible lags were produced for each participant, and maximum CC values were extracted, as well as the lag time at which the maximum CC was achieved. The convention was adopted that negative lag time values indicated the cardiovascular measure (SV, HR, TPR) lagged the muscle measure (EMG, NIRS) and positive lag time values indicated that the cardiovascular measure led the muscle measure. Means and ranges of CC values and lag times for the entire cohort were also calculated.

## 3 Results

**FIGURE 3:**
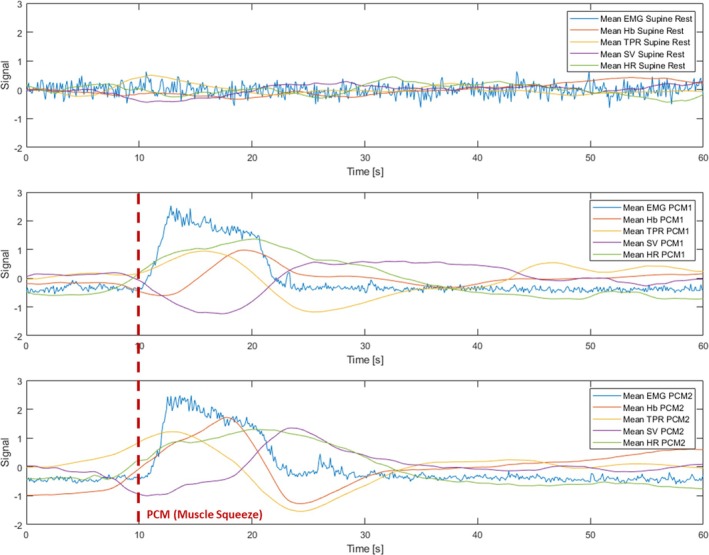
Mean plots of all normalised signals at each ROI in the 22 participants. Abbreviations: electromyography (EMG); total haemoglobin concentration (Hb); heart rate (HR); stroke volume (SV); total peripheral resistance (TPR); physical counterpressure manoeuvre (PCM).

The mean age of participants was 70.4 ± 5.2 years (range 59 to 82 years) and 77.3% were women. Additional descriptives for the sample are provided in Table 1. Figure 2 shows all mean signals in the 22 participants, including changes from baseline and the locations from which ROIs were extracted. Figure 3 shows the mean plots for all normalised signals at each ROI (supine rest, PCM1, and PCM2). Visually, there were marked differences between the supine rest signals and those acquired during either PCM. During supine rest, the signals appeared relatively stationary, with little to no visible trends, as would be expected (Figure 2, middle strip; Figure 3, top panel). Conversely, in signals acquired during either PCM (Figure 2, lateral strips; Figure 3, bottom panels), the 10 s squeeze (starting at 10 s in Figure 3 bottom panels) was very apparent in the EMG data, as were the changes in the other measured physiological signals.

**TABLE 1:** Demographic descriptives of the cohort (*N* = 22).

Age [years]	Mean (SD) 70.4 ± 5.2; range 59 to 82
**Sex [female]**	77.3%
**Body mass index (BMI) [kg/m** ^**2**^ **]**	Mean (SD) 26.2 ± 3.7; range 17.2 to 34.3
**Smoker**	16 Never; 6 past
**Alcohol consumption [units/week]**	Mean (SD) 7.0 ± 7.4; range 0 to 24
**Number of cardiovascular conditions**	
0	40.9%
1	27.3%
+2	31.8%
**On cardiovascular medications [yes]**	36.4%
**On psychotropic medications [yes]**	18.2%

The morphologies of the EMG and HR signals were similar during the supine (PCM 1) and standing (PCM 2) squeezes, even though, as shown in Figure 2, the baseline of both signals was higher in PCM 2 than in PCM 1.

For the other signals, response patterns between PCM 1 and PCM 2 seemed to differ. As regards haemoglobin concentration in the thigh, we observed that PCM 1 showed a more subdued response, which occurred later and only appeared to dip below baseline briefly at the initiation of the squeeze (Figure 3); in contrast, during PCM 2, from a much higher baseline (Figure 2), we observed a faster response, which raised before the squeeze, then dropped relatively sharply below baseline, and recovered approximately 20 s after the squeeze (Figure 3).

In addition, in PCM 2, TPR also displayed a higher baseline (Figure 2), was higher prior to the squeeze, and seemed to drop faster and deeper post‐squeeze (Figure 3).

SV responses seemed most different between PCM 1 and PCM 2. Figure 2 shows that SV was the only signal with a lower baseline post‐stand than pre‐stand. In Figure 3, we observed that SV had a slow decline and steady rise back to near baseline during PCM 1, whereas during the standing squeeze (PCM 2), the drop was much more pronounced but recovered sharply to beyond baseline as the squeeze ended.

Also of note, and as visualised in Figure 2, is that cardiac output (CO) and mean arterial pressure (MAP) increased on average by 1.0 L/min and 12 mmHg, respectively, during PCM 1, and by 1.0 L/min and 18 mmHg, respectively, during PCM 2.

Analyses of stationarity (Table S1) revealed that surface EMG signals were already covariance‐stationary in 100% of participants across all three conditions, permitting level‐data VAR modelling. In contrast, nearly all cardiovascular variables required transformation: TPR failed stationarity in 86.3–100% of cases, SV in 90.9–95.5% and HR in 86.4–95.5%, necessitating first‐order differencing. Post‐difference, 18.2–59.1% of TPR, 86.4–100% of SV and 81.8–95.5% of HR series met stationarity criteria. Thigh Hb exhibited intermediate behaviour: raw stationarity in just 27.3–31.8% of participants and post‐difference stationarity in 36.4–90.4% of cases.

**FIGURE 4:**
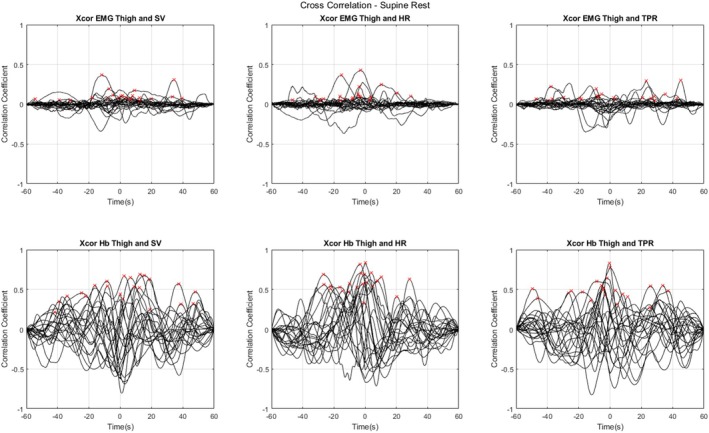
Individual cross‐correlation functions (CCFs) plotted against lag times in the 22 participants. Data acquired during supine rest. Individual maximum peaks are highlighted with a red ‘x’. Negative time values indicate that the cardiovascular measure (SV, HR, TPR) lags the muscle measure (EMG, NIRS), and positive time values indicate that the cardiovascular measure leads the muscle measure. Abbreviations: electromyography (EMG); total haemoglobin concentration (Hb); heart rate (HR); stroke volume (SV); total peripheral resistance (TPR).

**FIGURE 5:**
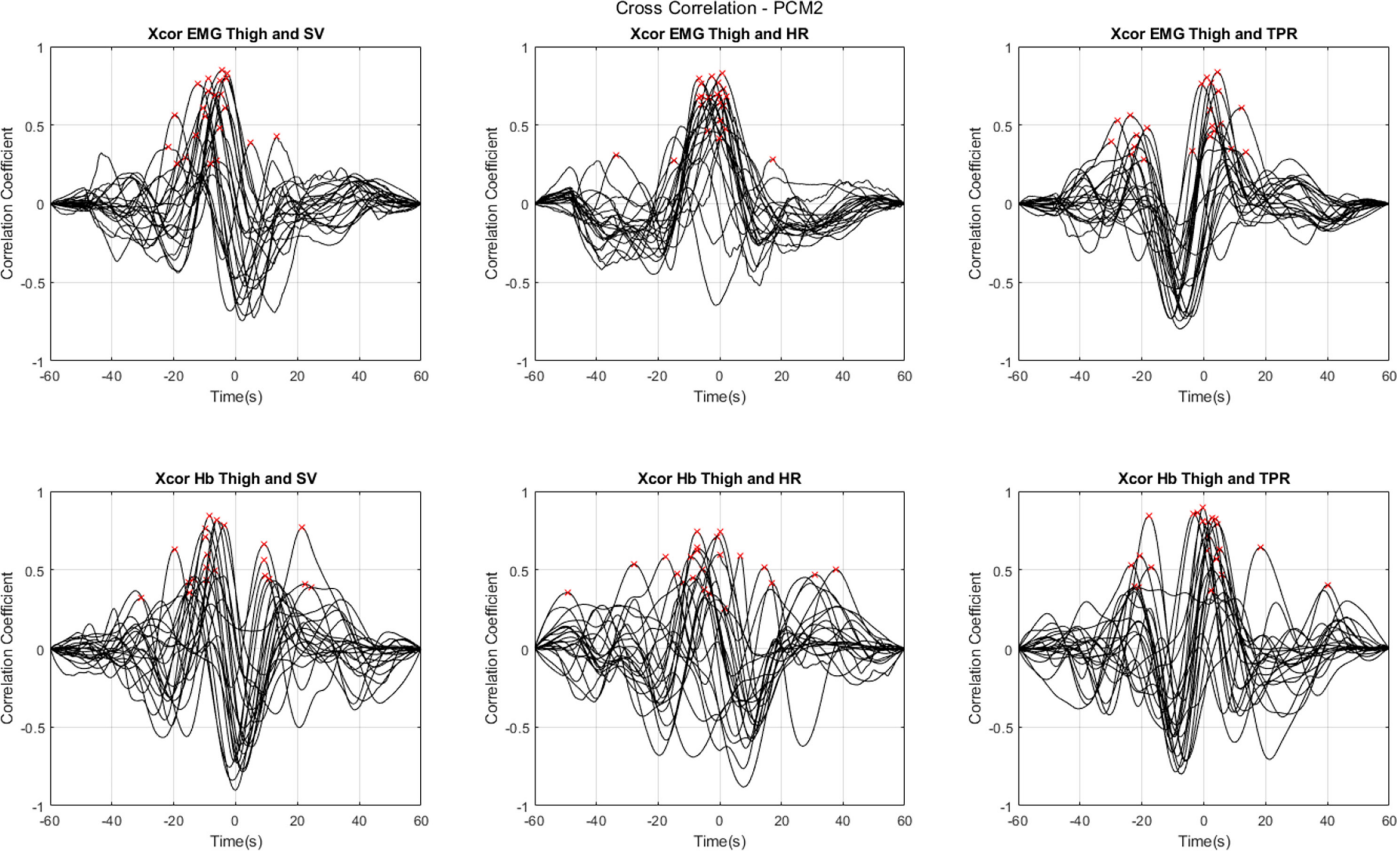
Individual cross‐correlation functions (CCFs) plotted against lag times in the 22 participants. Data acquired during physical counterpressure manoeuvre (PCM) 1 (supine). Individual maximum peaks are highlighted with a red ‘x’. Negative time values indicate that the cardiovascular measure (SV, HR, TPR) lags the muscle measure (EMG, NIRS), and positive time values indicate that the cardiovascular measure leads the muscle measure. Abbreviations: electromyography (EMG); total haemoglobin concentration (Hb); heart rate (HR); stroke volume (SV); total peripheral resistance (TPR).

**FIGURE 6:**
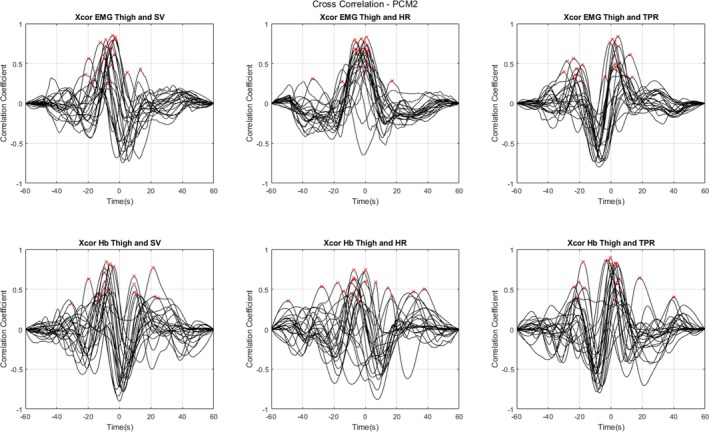
Individual cross‐correlation functions (CCFs) plotted against lag times in the 22 participants. Data acquired during physical counterpressure manoeuvre (PCM) 2 (standing). Individual maximum peaks are highlighted with a red ‘x’. Negative time values indicate that the cardiovascular measure (SV, HR, TPR) lags the muscle measure (EMG, NIRS), and positive time values indicate that the cardiovascular measure leads the muscle measure. Abbreviations: electromyography (EMG); total haemoglobin concentration (Hb); heart rate (HR); stroke volume (SV); total peripheral resistance (TPR).

The cross‐correlation analyses revealed significant temporal relationships between various physiological signals, as summarised in Table 2 and Supplemental 1 (Figure S1), and visualised in more detail in Figures 4, 5 and 6. The latter three figures illustrate the individual CCFs for specific signal pairs under different conditions (supine rest, PCM 1 and PCM 2, respectively), focusing on EMG and Hb as potential candidate markers of skeletal muscle pump, and highlighting the temporal dynamics of these relationships. Each individual participants' maximum peak CC values are also highlighted with a red ‘x’. These figures revealed a general trend in the data for lower CC values during supine rest compared with either PCM, for the EMG analyses; specifically, during supine rest, only 9.1% of observations had significant Granger causality with SV and HR, and 22.7% with TPR (Table 2; non‐stationary differenced method). However, while Hb was relatively weakly connected with TPR during supine rest (18.2%), it showed stronger connections with SV (54.4%) and HR (68.2%) during this phase.

**TABLE 2:** Results from cross‐correlation and Granger causality analysis.

	Cross‐correlation			
	Mean (range)	Mean (range)	Granger causality (non‐stationary differenced)	Granger causality (Toda–Yamamoto method)
	Peak Lag [s]	Peak CC	% Significant	% Significant
**Supine rest**				
EMG to SV	0.5 (−54.5 to 39.6)	0.111 (0.038 to 0.367)	9.1%	90.9%
EMG to HR	−8.6 (−46.5 to 29.2)	0.123 (0.044 to 0.428)	9.1%	100%
EMG to TPR	−0.5 (−47.2 to 45.2)	0.113 (0.048 to 0.299)	22.7%	18.2%
Hb to SV	−3.9 (−26.6 to 28.4)	0.484 (0.211 to 0.689)	54.5%	100%
Hb to HR	10.9 (−35.5 to 55.7)	0.589 (0.331 to 0.839)	68.2%	100%
Hb to TPR	3.3 (−41.7 to 48.1)	0.499 (0.272 to 0.830)	18.2%	100%
**PCM 1**				
EMG to SV	−10.3 (−35.0 to 11.7)	0.431 (0.060 to 0.802)	50.0%	95.5%
EMG to HR	−1.0 (−15.2 to 22.3)	0.597 (0.078 to 0.817)	18.2%	40.9%
EMG to TPR	−9.3 (−38.4 to 12.9)	0.537 (0.065 to 0.873)	40.9%	90.9%
Hb to SV	−5.5 (−41.4 to 31.8)	0.590 (0.356 to 0.895)	45.5%	100%
Hb to HR	7.3 (−12.9 to 39.0)	0.576 (0.076 to 0.913)	27.3%	100%
Hb to TPR	−5.0 (−44.4 to 25.3)	0.596 (0.295 to 0.847)	40.9%	100%
**PCM 2**				
EMG to SV	−7.8 (−21.9 to 13.4)	0.564 (0.251 to 0.849)	63.6%	95.5%
EMG to HR	−3.1 (−33.7 to 17.3)	0.609 (0.276 to 0.828)	36.4%	54.5%
EMG to TPR	−5.9 (−30.0 to 13.6)	0.516 (0.278 to 0.835)	40.9%	77.3%
Hb to SV	−3.0 (−49.4 to 37.7)	0.519 (0.257 to 0.742)	45.5%	100%
Hb to HR	8.2 (−3.5 to 39.7)	0.496 (0.267 to 0.834)	27.3%	95.5%
Hb to TPR	−3.0 (−30.6 to 24.4)	0.574 (0.318 to 0.844)	40.9%	100%

*Note:* Negative time values indicate that the cardiovascular measure (SV, HR, TPR) lags the muscle measure (EMG, NIRS), and positive time values indicate that the cardiovascular measure leads the muscle measure.

Abbreviations: CC, correlation coefficient; EMG, electromyography; Hb, total haemoglobin concentration; HR, heart rate; PCM, physical counterpressure manoeuvre; SV, stroke volume; TPR, total peripheral resistance.

During PCMs, as shown in Table 2, the most noticeable differences in causality proportions were the increases in EMG to SV for both PCM 1 (50.0%) and PCM 2 (63.6%). There were also more modest increases in EMG to HR (18.2% and 36.4%, respectively) and EMG to TPR (40.9% for both PCM 1 and PCM 2).

Looking at Hb, Table 2 shows that the proportion of significance for SV was of relatively similar magnitude across all three phases (54.4% and 45.5% for supine rest and both PCMs, respectively). However, for HR, there appeared to be a decoupling during the PCMs compared to supine rest, with 27.3% significant for PCMs versus 68.2% for supine rest; by contrast, TPR had increased significance during both PCMs (40.9% [in both cases] versus 18.2%).

In terms of lag analyses, for the main finding of EMG to SV, the cross‐correlation analysis showed mean peak CCs of 0.431 and 0.564 for PCM 1 and PCM 2, respectively, with mean lag times of −10.3 s and −7.8 s, meaning SV lags EMG, with shorter lag during PCM 2 (standing). When examining EMG to HR, the highest mean peak CCs were observed during PCM 1 and PCM 2, at 0.597 and 0.609, with −1.0 s and −3.1 s lag times, both respectively. This means that HR also lags EMG, but by less time than SV and especially for the supine PCM. For EMG to TPR, the analysis revealed mean peak CCs for PCM 1 and PCM 2 of 0.537 and 0.516, with peak lag times of −9.3 s and −5.9 s, respectively. These results suggest that similar to SV and HR, TPR also lags muscle activation, by approximately 6 to 9 s, with shorter lag during PCM 2 (standing).

The data presented in Table 2 suggest inter‐individual variability in the CC values and lag times. For instance, the peak CC for EMG to SV during PCM 1 ranged from 0.060 to 0.802, and 0.251 to 0.849 in PCM 2, indicating that although strong correlations were generally observed, their strength varied markedly among individuals. Similarly, lag times showed substantial differences; for example, the lag time for EMG to SV during PCM 1 ranged from −35.0 to 11.7 s, and that of PCM 2 from −21.9 to 13.4 s, reflecting considerable variability in the timing of peak correlations across participants.

## 4 Discussion

This study examined the temporal relationships between thigh muscle activation, thigh haemoglobin concentration, and cardiovascular parameters (SV, HR, TPR) in a sample of older adults during supine rest and two different PCMs. The main finding was that muscle activation as measured by surface EMG was strongly predictive of change in cardiovascular parameters during PCMs. Specifically, in terms of EMG to SV, the proportion of significant causality increased by approximately 500% during supine PCM and 600% during standing PCM compared to supine rest; for EMG to HR, causality increased by approximately 100% during supine PCM and 300% during standing PCM compared to supine rest; and for EMG to TPR, causality increased by approximately 80% during both supine PCM and standing PCM compared to supine rest.

Haemoglobin concentration in the thigh was found to be less specific to PCM, showing smaller changes during PCMs versus supine rest, when compared with EMG responses. Specifically, the causality from Hb to SV decreased by 9% during both PCMs compared to supine rest; causality from Hb to HR decreased by approximately 41% during both PCMs compared to supine rest; and causality from Hb to TPR increased by approximately 23% during both supine PCM and standing PCM compared to supine rest.

Our results indicate that muscle activation in the lower limb, when measured by surface EMG during PCMs, significantly predicts changes in SV, HR and TPR, with distinct temporal lags between these physiological signals. Specifically, during the supine PCM (PCM 1), the peak in HR occurred on average 1 s after the peak in EMG, followed by the peak in TPR at 9 s and the peak in SV at 10 s. In the standing PCM, the peak HR was observed at 3 s, the peak TPR at 6 s, and the peak SV at 8 s.

Our results are consistent with a recent semi‐systematic review and meta‐analysis [9], which examined the role and mechanisms of PCMs as a preventative intervention for vasovagal syncope. The review included 45 studies of various designs and settings and found that CO increased in most studies, with a small increase in HR, suggesting that the improvements in CO were likely driven largely by SV. TPR responses were variable, with some studies reporting increases in TPR and some reporting decreases, which were felt to be due to different PCM approaches and techniques across studies.

CO is defined as the product of SV and HR; and SV is intrinsically regulated by preload, which refers to the degree of stretch in the ventricles before they contract. An increase in the volume or speed of venous return (such as during PCMs) will elevate preload, and according to the Frank–Starling law, this will subsequently increase SV [29, 30]. Our results suggest that this was the main mechanism by which PCMs acted in our participants, and its longest time lag would be explained by the slower transit time of blood through the venous circulation, supporting the ‘muscle pump’ hypothesis [7, 31]. However, a direct increase in HR, which appeared to be the second most important mechanism, could be rapidly triggered by voluntary muscle contraction by virtue of the small muscle fibre mechanoreceptors responding to stretch, inhibiting cardiac vagal activity and thus rapidly increasing HR [32, 33] (e.g., within 1–3 s as seen in our results). In addition, PCMs may increase TPR [34] (third most important mechanism in our sample) via the slower muscle sympathetic nerve activity (MSNA) [35]. Ultimately, the product of CO and TPR will determine the rise in blood pressure during the PCMs [36]. Overall, the distinct lag times for SV, HR, and TPR highlight the sequential nature of these interactions, supporting the concept that the skeletal muscle pump operates through a cascading effect on cardiovascular function [37].

During PCM testing, all cardiovascular measures lagged all muscle measurements from approximately 1 to 10 s, except for Hb to HR, where thigh Hb consistently lagged HR by approximately 7 to 11 s. In fact, this relationship remained as strong during supine rest, and under that condition, participants had the highest proportion of significance for Hb to HR compared with other measures tested. This could be due to close coupling of coherent oscillations due to the cardiac cycle present both in HR and Hb series. Indeed, this close coupling between thigh haemoglobin concentration and cardiovascular measures may be why there was not a great difference in cross‐correlation analysis and Granger causality results between supine rest and either PCM for NIRS‐based analysis. Conversely, EMG‐based analysis provided very good differentiation between supine rest and both PCMs using the correlation and causality analysis framework presented herein.

It is also worth noting that although similar HR responses were observed during both supine and standing PCMs, the SV response differed, as shown in Figure 3, with the standing muscle squeeze showing a faster and more dynamic change in SV. In parallel with this, we also observed distinctly different patterns in thigh Hb response, again with the standing squeeze resulting in a much faster and more dynamic response, with the faster reduction in Hb (after an initially faster increase) occurring at approximately the same time as SV increased. This may reflect an interplay between muscle contraction‐induced blood expulsion and compensatory vasoconstriction mechanisms and suggests that gravitational forces intensify the reliance on the muscle pump for maintaining adequate venous return and CO. These observations align with previous studies indicating that the baroreflex‐mediated adjustments in vascular tone are more pronounced during upright posture due to increased orthostatic stress [38]. These distinct responses in SV and thigh Hb during standing PCM (versus supine PCM) could potentially provide a method for non‐invasive investigation of skeletal muscle pump function which warrants further study; in such studies, contraction intensity could be systematically varied—perhaps using real‐time EMG feedback to titrate force—and measure the resulting changes in cardiovascular measures to define the optimal ‘dose’ of PCM for preventing orthostatic intolerance. Age‐related loss of skeletal muscle mass and function—sarcopenia—can impair the muscle pump and is highly prevalent in older adults. Moreover, given that sarcopenia has been linked to higher risks of both depressive symptoms and cardiovascular events in large longitudinal cohorts [39, 40], interventions aimed at preserving or augmenting lower‐limb muscle pump function—such as targeted PCMs—may confer broader benefits for mental health and CVD prevention in older adults.

A key strength of this study is the use of multimodal data collection, incorporating surface EMG, NIRS and non‐invasive beat‐to‐beat cardiovascular measurements, which enabled a thorough analysis of the complex interactions between muscle activation and cardiovascular responses. The application of advanced signal processing techniques, such as cross‐correlation and Granger causality analysis, allowed for the identification of significant temporal relationships and predictive dynamics with high precision. This study also benefited from a well‐defined experimental protocol and rigorous data collection and processing methods, enhancing the reliability and internal validity of the findings.

To ensure that Granger‐causality findings were statistically valid, it was first confirmed that each time series met the requirement of covariance stationarity by applying both the ADF and KPSS tests to every ROI signal (EMG, Hb, SV, HR, TPR) before fitting any models. As shown in Table S1, EMG was inherently stationary (100% of participants), allowing level‐data VAR modelling, whereas HR and SV required first‐order differencing in most cases (with 82–100% achieving stationarity post‐difference). Hb and TPR showed mixed differencing success (18–90% across ROIs). To guard against any residual bias from pre‐differencing—especially in the Hb and TPR analyses—we complemented this with the Toda–Yamamoto procedure. Both approaches converged on the central insight that both thigh EMG and Hb robustly drive changes in SV, HR, and TPR during PCMs—though the level‐data Toda–Yamamoto tests yielded higher levels of significance overall, highlighting their sensitivity to long‐run coupling that differencing may attenuate. This divergence aligns with our stationarity checks: differencing removed long‐run trends before standard Granger tests, whereas the augmented VAR retained them, thus boosting level‐data significance. By integrating these complementary strategies—rigorous stationarity testing, conservative differenced‐VAR, and augmented‐VAR causality—we offer a transparent, replicable framework for physiological time‐series analysis that balances statistical rigour with full‐spectrum dynamic sensitivity.

Despite these strengths, the study has several limitations. Even though the 95% confidence intervals for the mean signals depicted in Figure 2 are quite narrow, especially during the supine rest, the small sample size (*n* = 22) may have contributed to the observed inter‐individual variability, especially during the performance of PCMs. The data presented in Table 2 suggest inter‐individual variability in the CC values and lag times. For instance, the peak CC for EMG to SV during PCM 1 ranged from 0.060 to 0.802, and 0.251 to 0.849 in PCM 2, indicating that although strong correlations were generally observed, their strength varied markedly among individuals. Similarly, lag times showed substantial differences; for example, the lag time for EMG to SV during PCM 1 ranged from −35.0 to 11.7 s, and that of PCM 2 from −21.9 to 13.4 s, reflecting considerable variability in the timing of peak correlations across participants. The variability in causality significance and lag times across participants possibly indicates inter‐individual differences in the efficiency of muscle pump function, which could be influenced by factors such as muscle strength, vascular health, and overall cardiovascular fitness.

The small sample size also precluded subgroup analyses. For example, with only five men in the sample, sub‐analysis by sex was not pursued. In terms of age, the cohort was primarily composed of older adults with a mean age of 70 years, and it was not possible to establish a comparison with a younger group. With a larger sample size and more diverse demographics and clinical features, it would have been possible to explore predictors of interindividual variation, and further research could utilise the methodology to focus on identifying specific demographic, physiological or health‐related factors that influence the effectiveness of the muscle pump during PCMs. This could facilitate the refinement and personalisation of effective interventions to manage and prevent OH.

Furthermore, our PCMs were in the lower limbs, and upper limb PCMs, which have also been reported in the literature [41], were not part of our study design. Additionally, we only recorded EMG/NIRS from the primary thigh muscles, without monitoring adjacent or antagonist muscle groups (e.g., gluteals, hip adductors). Consequently, any unmeasured co‐contractions may have contributed to the variability in both EMG/NIRS amplitude and the timing of hemodynamic responses, potentially confounding our estimates of muscle‐pump efficiency. Future studies should include multi‐site EMG recordings to quantify co‐activation patterns and better isolate the specific contribution of the targeted muscle group to cardiovascular changes during PCMs. We also did not record respiratory activity or control for central motor drive during the PCMs. Although the protocol was designed to minimise abrupt changes, voluntary contraction of the thigh muscles likely involved central command, which can independently elevate heart rate prior to and during the manoeuvre, and unmonitored breath‐holding or Valsalva‐like efforts could have further influenced cardiovascular response. Future studies could include simultaneous respiratory monitoring (e.g., pneumotachography) or implement controlled breathing protocols—and could even compare with electrically‐stimulated contractions—to disentangle purely mechanical muscle‐pump effects from centrally mediated cardiovascular responses.

It is also worth noting that the arterial baroreflex and other intrinsic cardiovascular control systems can act as dynamic filters—high‐pass or low‐pass—influencing both the timing and magnitude of signal correlations [42]. These reflex‐mediated filters may shift the peaks and lags detected by cross‐correlation and could bias Granger causality estimates by attenuating or delaying certain frequency components. While our use of cross‐correlation and Granger causality provides complementary insights into directionality, future studies should include direct measures of baroreflex sensitivity (e.g., sequence method or transfer‐function analysis) or employ pharmacological blockade to quantify and account for these reflex filtering effects on muscle‐pump–cardiovascular interactions.

Overall, our study focused solely on the haemodynamic mechanisms of lower‐limb PCMs, rather than their effectiveness in modifying blood pressure or reducing the incidence of OH or falls in our participants. Addressing these outcomes would have required a longitudinal design. Nevertheless, existing literature already provides evidence that PCMs are a risk‐free, effective and low‐cost treatment method for patients with vasovagal syncope and recognisable prodromal symptoms. They should be recommended as a first‐line treatment for patients presenting with vasovagal syncope with prodromal symptoms [43], although other strategies may be needed in addition to PCMs for the prevention of adverse outcomes in clinical populations [44].

In conclusion, our study reported a novel methodology that, by integrating multimodal non‐invasive monitoring techniques with cross‐correlation and Granger causality analyses, could enhance our ability to predict and model dynamic and time‐lagged cardiovascular responses to PCMs. Based on our results, we recommend that surface EMG be used preferentially to thigh NIRS when association with cardiovascular parameters during PCMs is of primary interest. This method could also lead to the development of a clinically useful measure of skeletal muscle pump function, and thereby a method to track any negative alterations to this physiological mechanism as we age or in the presence of disease that could impair muscle function. By enhancing our understanding of the temporal relationships between muscle activation and cardiovascular responses, this research could facilitate the future development of effective interventions to improve cardiovascular health and reduce the risk of OH and falls in older adults.

## Ethics Statement

Ethical approval for the study was granted from the Tallaght University Hospital/St. James's Hospital Joint Research Ethics Committee (Project ID: 0221; approval date: 4 May 2021; Dublin, Ireland) and approval was also granted by St James's Hospital Research & Innovation Office (Reference: 6567, approval date: 26 July 2021; Dublin, Ireland). All participants in the study provided explicit, written, informed consent. The study adhered to the World Medical Association Declaration of Helsinki on ethical principles for medical research involving human subjects.

## Conflicts of Interest

The authors declare no conflicts of interest.

## Supporting information


**Figure S1.** Illustration of the directionality of lag, as well as mean correlation coefficient (CC), mean lag time and the proportion of participants who had significant Granger causality results at that specific lag (from non‐stationary differenced method). Shown for all three experimental paradigms: supine rest, physical counter‐manoeuvre (PCM) 1 (supine) and PCM 2 (standing). Negative lag time values (green arrows) indicate that the cardiovascular measure (SV, HR, TPR) lags the muscle measure (EMG, NIRS), and positive time values (orange arrows) indicate that the cardiovascular measure leads the muscle measure. Abbreviations: electromyography (EMG); total haemoglobin concentration (Hb); heart rate (HR); stroke volume (SV); total peripheral resistance (TPR).

**Table S1.** Results from augmented Dickey–Fuller (ADF) and Kwiatkowski–Phillips–Schmidt–Shin (KPSS) test of stationarity, both pre‐ and post‐differencing.
