# Supplementary material for: Assessing the Skeletal Muscle Pump During Lower Limb Counterpressure: Lags and Causality in Cardiovascular Regulation
Source: J Cachexia Sarcopenia Muscle. 2025 Jul 31;16(4):e70019. doi: 10.1002/jcsm.70019 (PMC12311617; doi:10.1002/jcsm.70019)
Supplement: Supplementary file 1 — Figure S1. Illustration of the directionality of lag, as well as mean correlation coefficient (CC), mean lag time and the proportion of participants who had significant Granger causality results at that specific lag (from non‐stationary differenced method). Shown for all three experimental paradigms: supine rest, physical counter‐manoeuvre (PCM) 1 (supine) and PCM 2 (standing). Negative lag time values (green arrows) indicate that the cardiovascular measure (SV, HR, TPR) lags the muscle measure (EMG, NIRS), and positive time values (orange arrows) indicate that the cardiovascular measure leads the muscle measure. Abbreviations: electromyography (EMG); total haemoglobin concentration (Hb); heart rate (HR); stroke volume (SV); total peripheral resistance (TPR). [file JCSM-16-e70019-s001.docx]

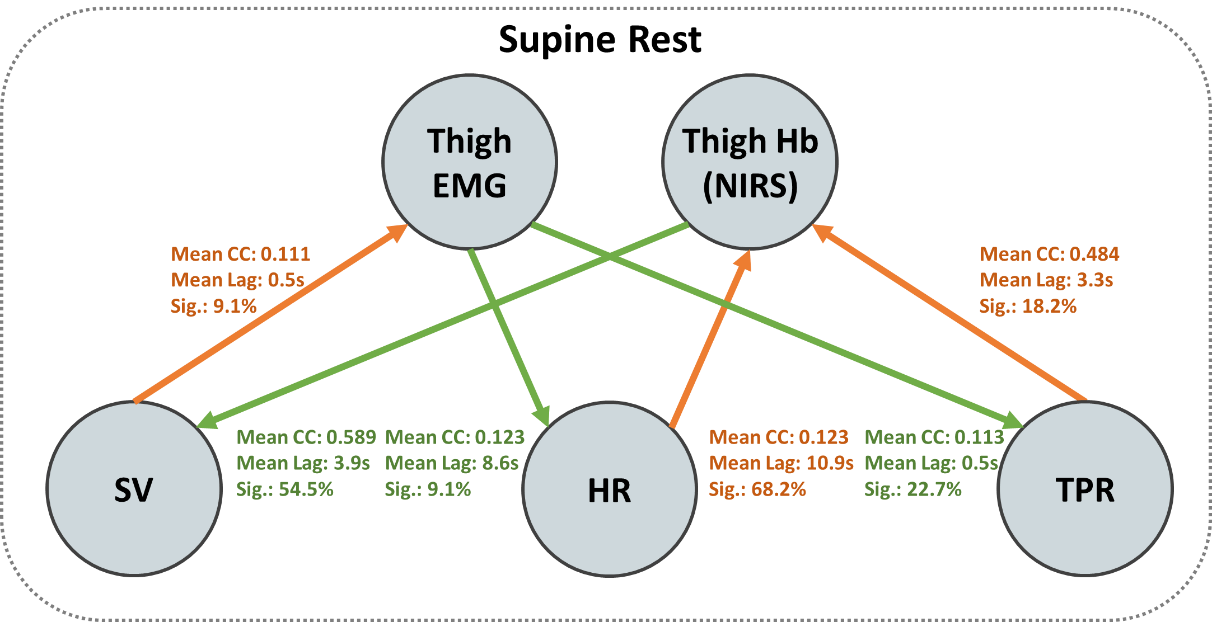


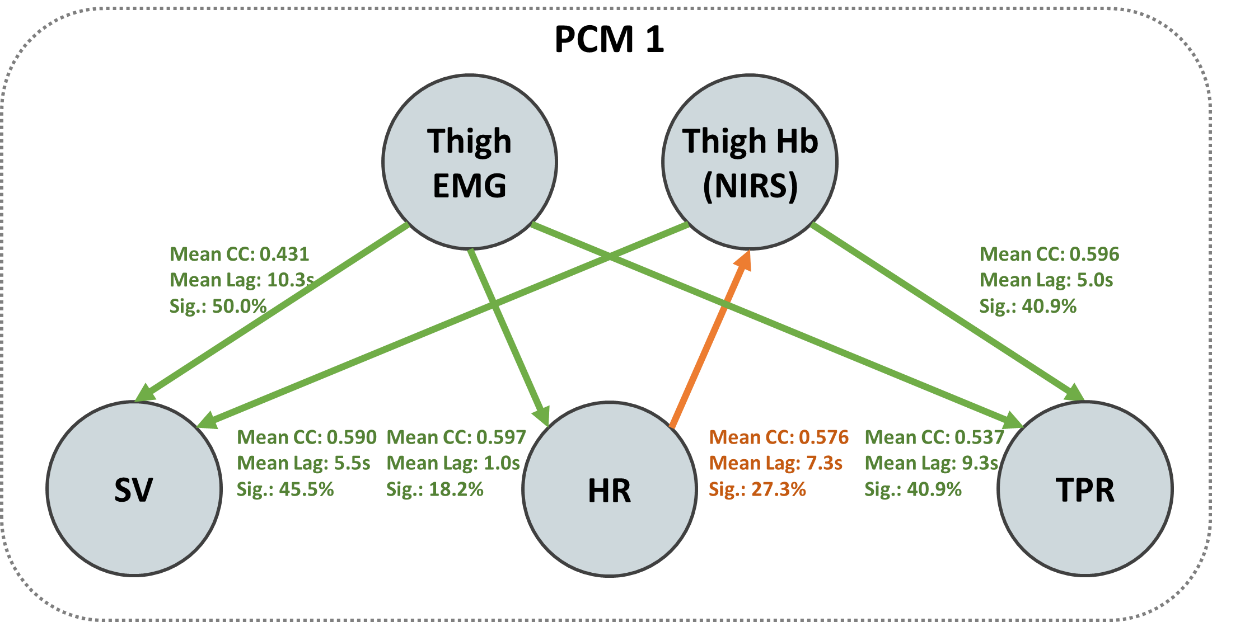


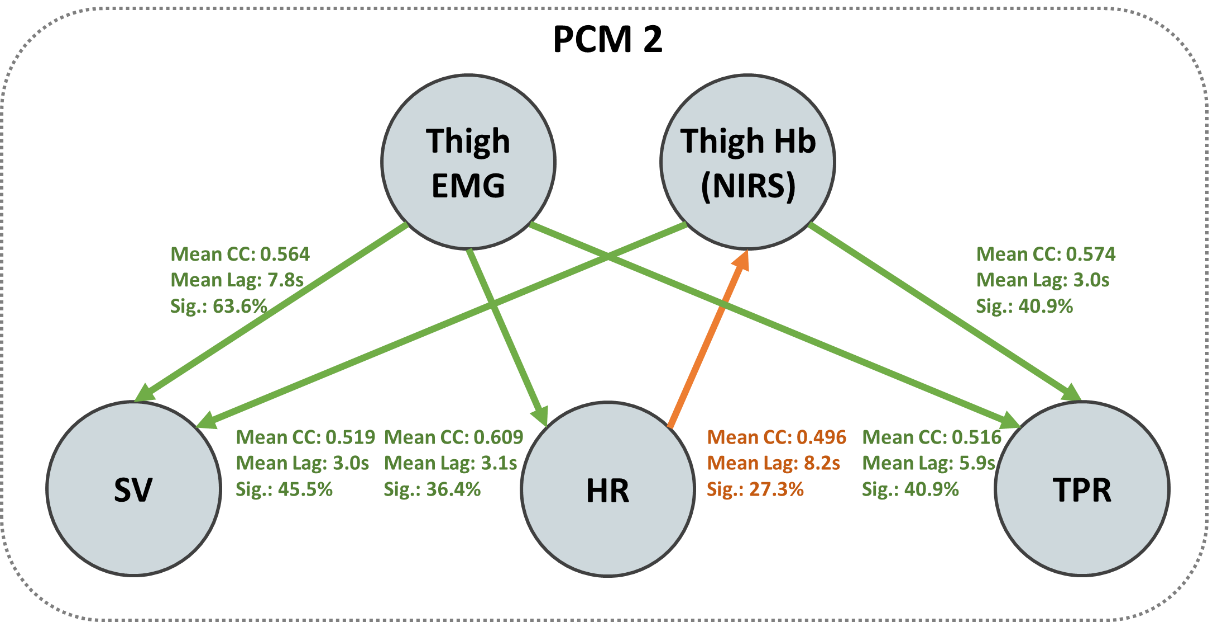


**Fig. S1** Illustration of the directionality of lag, as well as mean correlation coefficient (CC), mean lag time, and the proportion of participants who had significant Granger causality results at that specific lag (from non-stationary differenced method). Shown for all three experimental paradigms: supine rest, physical counter-manoeuvre (PCM) 1 (supine), and PCM 2 (standing). Negative lag time values (green arrows) indicate that the cardiovascular measure (SV, HR, TPR) lags the muscle measure (EMG, NIRS), and positive time values (orange arrows) indicate that the cardiovascular measure leads the muscle measure. Abbreviations: electromyography (EMG); total haemoglobin concentration (Hb); heart rate (HR); stroke volume (SV); total peripheral resistance (TPR)
