# Supplementary material for: Assessing the Skeletal Muscle Pump During Lower Limb Counterpressure: Lags and Causality in Cardiovascular Regulation
Source: J Cachexia Sarcopenia Muscle. 2025 Jul 31;16(4):e70019. doi: 10.1002/jcsm.70019 (PMC12311617; doi:10.1002/jcsm.70019)
Supplement: Supplementary file 2 — Table S1. Results from augmented Dickey–Fuller (ADF) and Kwiatkowski–Phillips–Schmidt–Shin (KPSS) test of stationarity, both pre‐ and post‐differencing. [file JCSM-16-e70019-s002.docx]

**Table S1** Results from Augmented Dickey–Fuller (ADF) and Kwiatkowski–Phillips–Schmidt–Shin (KPSS) test of stationarity, both pre- and post-differencing.

|  | % Stationary RAW data | % Stationary after differencing |
| --- | --- | --- |
| **Supine Rest** |  |  |
| EMG | 100% | N/A |
| Hb | 27.3% | 90.4% |
| HR | 13.6% | 95.5% |
| SV | 9.1% | 100% |
| TPR | 13.6% | 59.1% |
| **PCM 1** |  |  |
| EMG | 100% | N/A |
| Hb | 27.3% | 40.9% |
| HR | 13.6% | 81.8% |
| SV | 9.1% | 86.4% |
| TPR | 9.1% | 36.4% |
| **PCM 2** |  |  |
| EMG | 100% | N/A |
| Hb | 31.8% | 36.4% |
| HR | 4.5% | 90.9% |
| SV | 4.5% | 86.4% |
| TPR | 0% | 18.2% |

Abbreviations: electromyography (EMG); total haemoglobin concentration (Hb); heart rate (HR); stroke volume (SV); total peripheral resistance (TPR); physical counterpressure maneuver (PCM).
